# Integrated analysis to identify the prognostic and immunotherapeutic roles of coagulation-associated gene signature in clear cell renal cell carcinoma

**DOI:** 10.3389/fimmu.2023.1107419

**Published:** 2023-03-17

**Authors:** Guicao Yin, Tai Tian, Xing Ji, Shengqi Zheng, Zhenpeng Zhu, Yifan Li, Cuijian Zhang

**Affiliations:** ^1^ Affiliated Hospital of Yangzhou University, Yangzhou University, Yangzhou, China; ^2^ Department of Urology, Peking University First Hospital, Beijing, China; ^3^ Department of Urology, The Third Affiliated Hospital of Hebei Medical University, Shijiazhuang, China

**Keywords:** coagulation-related gene, clear cell renal cell carcinoma, diagnostic signature, prognostic signature, individualized treatment

## Abstract

The coagulation system is closely related to the physiological status and immune response of the body. Recent years, studies focusing on the association between coagulation system abnormalities and tumor progression have been widely reported. In clear cell renal cell carcinoma (ccRCC), poor prognosis often occurs in patients with venous tumor thrombosis and coagulation system abnormalities, and there is a lack of research in related fields. Significant differences in coagulation function were also demonstrated in our clinical sample of patients with high ccRCC stage or grade. Therefore, in this study, we analyzed the biological functions of coagulation-related genes (CRGs) in ccRCC patients using single-cell sequencing and TCGA data to establish the 5-CRGs based diagnostic signature and predictive signature for ccRCC. Univariate and multivariate Cox analyses suggested that prognostic signature could be an independent risk factor. Meanwhile, we applied CRGs for consistent clustering of ccRCC patients, and the two classes showed significant survival and genotype differences. The differences in individualized treatment between the two different subtypes were revealed by pathway enrichment analysis and immune cell infiltration analysis. In summary, we present the first systematic analysis of the significance of CRGs in the diagnosis, prognosis, and individualized treatment of ccRCC patients.

## Introduction

The ccRCC is the major pathologic subtype of kidney cancer (1). Untimely first diagnosis and postoperative recurrence often lead to a poor prognosis (2, 3). As a vigorously immunogenic tumor with the properties of insensitivity to radiotherapy and chemotherapy, there is growing evidence of the therapeutic value of immune checkpoint inhibitors (ICIs) in ccRCC (4, 5). However, due to the heterogeneity of ccRCC and different tumor microenvironments (TME), the application of ICIs is still limited (6). Therefore, it is particularly important to explore the relevant factors impacting the TME and thus ICIs in ccRCC.

The coagulation system is critical for innate defense mechanisms and is strongly associated with the TME of ccRCC. Numerous experimental data suggest that patients with malignancies have chronic hypercoagulation and hyper fibrillation (7). Interestingly, the process of cancer development theoretically necessitates a large blood supply, however, the patient with a tumor is 9-fold more likely to develop cancer-related thrombosis than the healthy (8–10). In recent years, studies on coagulation and tumor ICI seem to present different outcomes (11, 12). Nevertheless, the coagulation system is sophisticated and complex and needs to be analyzed in a systematic manner. In ccRCC, there seemed to be some synergistic link between coagulation and inflammation (13, 14). Therefore, focusing on the role of coagulation-related genes (CRGs) in ccRCC might support prognostic evaluation and ICIs treatment.

In addition, with the development of genome sequencing technology, increasingly patients could benefit from the individualized genomic treatments (15). Clinicians have shifted to the use of bioinformatics to discover biomarkers and molecular processes in different diseases. Currently, several prognostic signatures have been established in ccRCC to predict the prognosis of patients (16, 17). However, there is still a lack of an effective signature to evaluate the therapeutic effect, which needs to be explored.

Therefore, in this study, we first systematically analyzed the expression and prognostic value of CRGs in ccRCC. Then, we classified the patients into different coagulation statuses based on consistent clustering and examined the differences of immune infiltration, biological difference, and therapeutic choice between clusters. Then we constructed 5-CRG based diagnostic signature and 8-CRG based prognostic signature. The AUC value of ROC curve shows good diagnostic and predictive efficiency. Afterward, combined with independent clinical risk factors, we constructed a predictive nomogram. Finally, we validated FDX1 in clinical samples and cell lines.

## Materials and methods

### Data processing

The transcriptome profile and corresponding clinical information for ccRCC samples were downloaded from The Cancer Genome Atlas (TCGA-KIRC, http://protal.gdc.cancer.gov/). Furthermore, we obtained the validation cohort with follow-up information from the ArrayExpress database (E-MTAB-1980, https://www.ebi.ac.uk/arrayexpress/). For the data format, the fragments per kilobase million (FPKM) were transformed into transcripts per million (TPM). Further, the ccRCC and normal kidney single cell sequencing data were downloaded from the GEO database (GSE159115). We used Seurat v4 to process single cell data and merge them. Afterward, we annotated the different clusters according to the marker genes reported in the previous studies. Also, we collected preoperative coagulation data from the case system for ccRCC patients from the First Affiliated Hospital of Yangzhou University, and all protocols met the requirements of the ethics committee of Yangzhou University.

### Molecular subtyping and therapeutic prediction

In order to classify ccRCC patients for personalized treatment, we performed molecular subtyping based the CRGs using the *ConsensusClusterPlus* R package with the follow setings (maxK=7, reps=100, pItem=0.8, pFeature=1, distance=“manhattan”, clusterAlg=“pam”) (18). Meanwhile, unsupervised clustering and the corresponding representative data were generated using the *ggplot2* R package. To explore the immune cell infiltrations between two clusters, the single sample gene set enrichment analysis (ssGSEA) was performed and each type of immune cell was calculated according to the score. After that, the effect of immunotherapy between different clusters were evaluated using the Cancer Immune Atlas (TCIA) database. The potential molecular enrichment between the clusters was annotated with the ClueGO plugin from the Cytoscape software. We then used the *pRRophetic* R package to assess of the sensitivity between different clusters to clinical drugs in advanced ccRCC (19).

### Identification of DECRGs and prognostic CRGs

First, we retrieved the CRGs from the MsigDB database (HALLMARK_COAGULATION and MALLMARK_COMPLEMENT, http://software.broadinstitute.org/gsea/msigdb), and the detailed information on the 281 CRGs were shown in the **Supplementary Table S1** (20). Then, in the R environment, the *limma* R package was condemned to screen out the differentially expressed CRGs (DECRGs) between ccRCC and normal samples, based on the set cutoff criteria of P<0.05 in TCGA and single-cell sequencing cohorts (21). Univariate Cox regression was used to identify the prognostic CRGs. Genes with the P value less than.05 in Cox regression were identified as prognostic genes for further LASSO regression analysis.

### Sample collection and quantitative PCR

The human RCC (786-O, 769-P, A498, ACHN, Caki-1, OS-RC-2, RCC4) and normal kidney cell lines (293) were cultured in Dulbecco’s modified eagle’s medium or RPMI 1640 with 10% fetal bovine serum and 1% Penicillin/Streptomycin. The cell lines were placed in a 37°C aseptic incubator with 5% CO_2_,and the fluid was changed every 2-3 days. The cell precipitation was collected, and the total RNA was extracted by TRIzol Reagent (Invitrogen). The patient samples were collected from the First Affiliated Hospital of Yangzhou University, and all the procedures were approved by the Ethics Committee. Then, cDNA synthesis was reverse transcribed using the Takara reagent kit. Then we performed quantitative PCR through SYBR green SuperMix and calculated the results using 2-ΔΔCT method (22). The primers used in this study can be found in **Supplementary Table S2**.

### Construction and validation of coagulation-related gene signature

To explore the prognostic value of the CRGs in ccRCC patients, we performed the LASSO regression of overall survival (OS) with a maxit=1000, using the *glmnet* R package. We divided the ccRCC samples in TCGA into training and validation cohorts according to the ratio of 3:2 and used the samples of E-MTAB-1980 as the external validation cohort. Then we calculated the risk score of each patient using the following formula: risk score = coffiCRG1 × CRG1 expression + coffiCRG2 × CRG2 expression + · ···· + coffiCRGn × CRGn expression. Afterwards, we used the *timeROC* R package to draw the patient’s 1-, 3-, and 5-year ROC curve to evaluate the prognostic value of the signature. At the same time, we used the *survival* R package and Kaplan-Meier ‘s method to compare the prognostic differences between high- and low-risk patients. Finally, through the univariate and multivariate regression analyses, we established a prognostic nomogram integrating the risk score and independent clinical parameters.

### Statistical analysis

All statistical analyses were conducted using R 4.1.3 and Prism GraphPad 9.3. Continuous variables were compared by using Student’s t test, the Mann–Whitney test, or the Wilcoxon rank-sum test. Meanwhile, cumulative survival analyses were performed using the Kaplan–Meier method, and the survival differences were analyzed using the log-rank test by *survival* R package. The predictive value of signature was evaluated using ROC cures. An AUC value greater than 0.75 is considered well, and a value greater than 0.60 is considered acceptable. The univariate and multivariate Cox regression were used to assess the correlation of the signature and clinical parameters with overall survival. Among all the results, P < 0.05 was considered to be statistically significant.

## Results

### Identification of DECRGs in ccRCC patients

**Figure 1:**
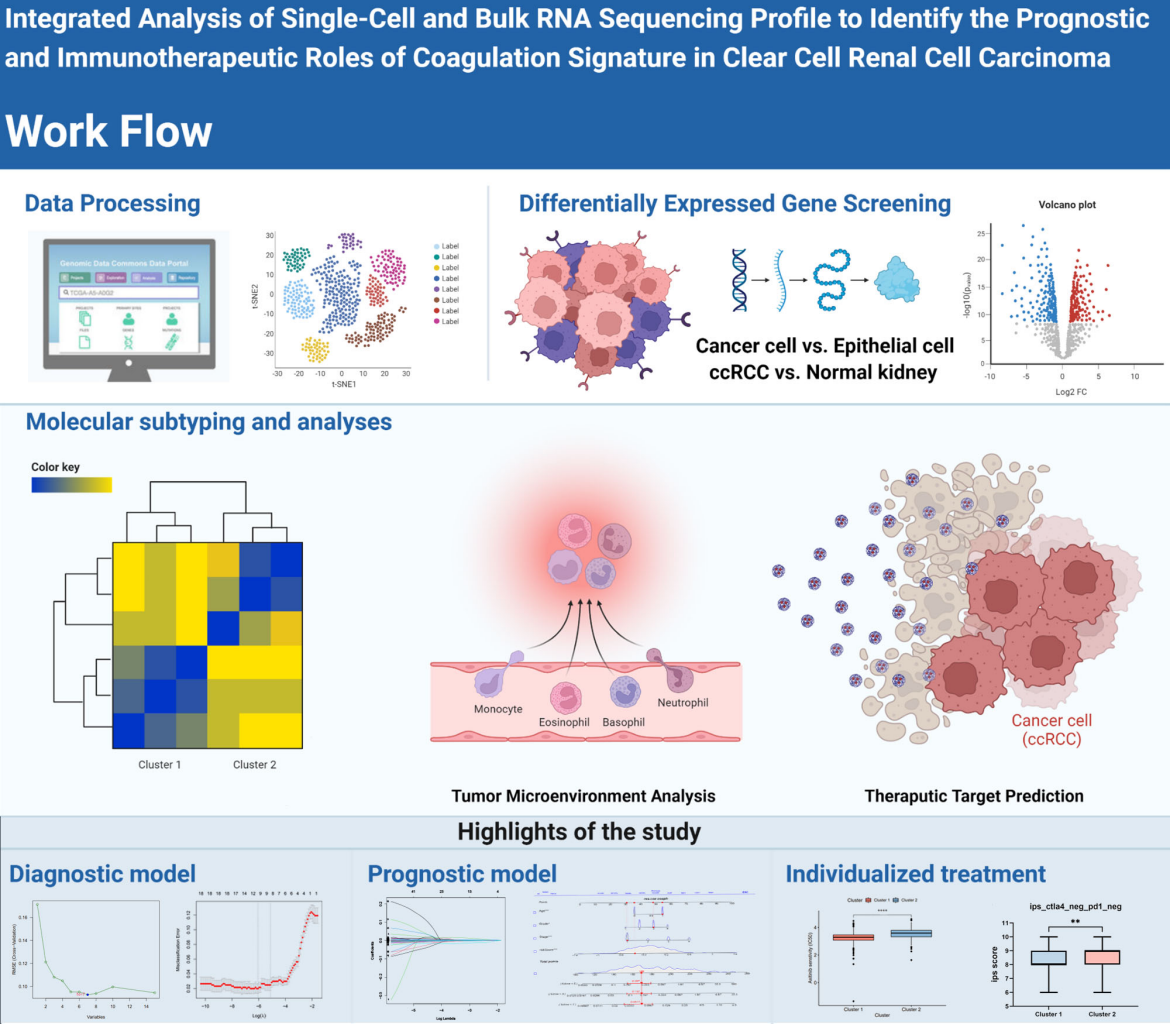
The graphical outline diagram of the whole process of this study. GP style: *: p< 0.05; **: p < 0.01; ***:p<0.001; **** p<0.0001.

**Figure 2:**
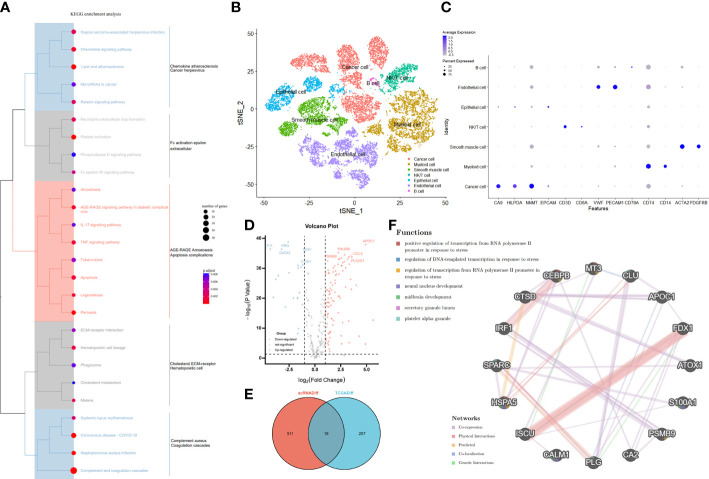
Identification and functional enrichment of differentially expressed CRGs. **(A)** The KEGG Tree enrichment plot of the 279 CRGs. **(B)** Reduced dimensional plots of tSNE for different cell types for single cell data. **(C)** Feature dot plots of different cell types for single cell data. **(D)** The Volcano map on differentially expressed CRGs in TCGA cohort. The five most significant up- and down-regulated genes were labeled separately. **(E)** VENN plots of the same differential genes in the Single Cell sequencing and TCGA cohorts. **(F)** PPI network created by GeneMANIA showing the interactions of the CRG.

First, the workflow for the whole study is shown in **Figure 1**. In our clinical work, we have found that patients with high stage or grade tend to be accompanied by more pronounced coagulation system dysfunction, which seems to be an interesting direction (**Supplementary Figure S1**). Based on the clinical founding and gene set of HALLMARKS, we obtained a total of 279 expression matrix of CRGs in the TCGA database. These CRGs were analyzed by KEGG Tree, and the functions of these CRGs were divided into a total of five broad categories, including apoptosis, coagulation, and important pathways such as complement and cytokines (**Figure 2A**). Subsequently, we performed a cluster analysis of the ccRCC single cell data (GSE159115) and annotated the different Clusters (**Figure 2B**), and the Dot plot of the relevant features is also shown in **Figure 2C**. Subsequently, we identified copy number variants in epithelial cells by the SCEVAN method and classified normal epithelial cells and malignant epithelial cells. By performing differential expression analysis between the two types of epithelial cells, we obtained a total of 527 differentially expressed genes with p <0.05. Meanwhile, the CRGs in TCGA data were analyzed by Limma package, and a total of 223 DECRGs were obtained with p <0.05 (**Figure 2D**). The DEGs from the single cell sequencing data were subsequently intersected with the DECRGs from TCGA to obtain a total of 16 hub genes (**Figure 2E**). We then performed correlation and regulatory pathway analysis by GeneMANIA on 16 hub genes, which are mainly involved in transcriptional regulatory functions in response to stress as well as coagulation-related mechanisms (**Figure 2F**).

### Molecular subtyping of ccRCC and therapeutic difference screening

**Figure 3:**
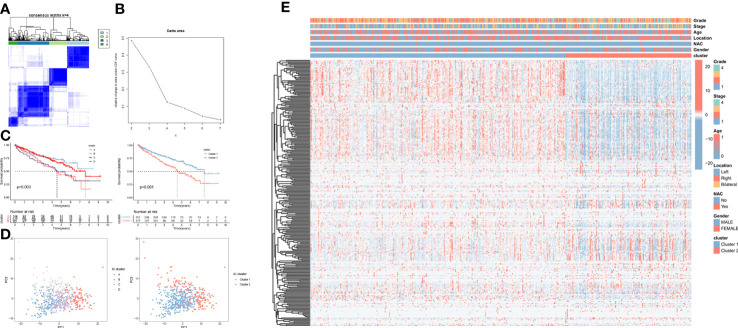
Molecular subtyping of ccRCC patients based on the CRGs in TCGA cohort. **(A)** The ccRCC patients were identified into 4 clusters according to the consensus clustering matrix (k = 4). **(B)** Relative changes in the area under the CDF curve by group number (MaxK = 7). **(C)** Kaplan–Meier survival curves for the 2 clusters and 4 clusters. **(D)** Principal component analysis for the 2 clusters and 4 clusters. **(E)** The heatmap and the clinical parameters of the 2 clusters established based on the CRGs.

To further investigate whether CRGs-based treatment can be individualized for ccRCC patients. We performed consistent clustering of ccRCC patients in TCGA based on 279 CRGs and initially classified patients into 4 clusters based on the decay of CDF values (**Figure 3A, B**). By performing survival analysis, the four Clusters showed obvious two survival states, so we merged Cluster A and Cluster C into Cluster 1 and Cluster B and Cluster D into Cluster 2. The two Clusters showed obvious survival differences between them (**Figure 3C**). It could also be seen by principal component analysis that when the TCGA samples are divided into 2 Clusters, the samples can be clearly separated, while with 4 Clusters, the boundaries of sample separation are not obvious (**Figure 3D**). The subsequent construction of a heatmap also demonstrated the existence of significant changes in gene expression profiles between these two Clusters (**Figure 3E**).

**Figure 4:**
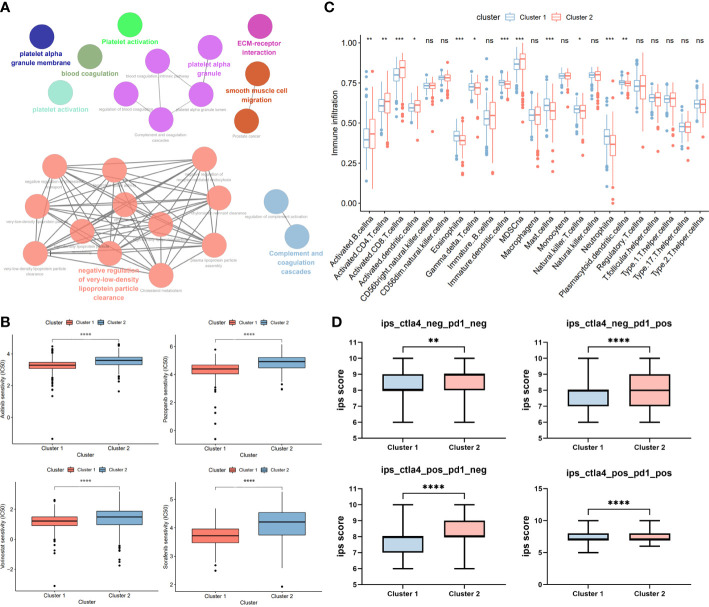
Phenotypic differences between clusters and potential individualized treatment. **(A)** The biological functional enrichment of differentially expression gene between two clusters in ClueGO plugin. **(B)** Drug sensitivity of commonly clinical used drugs for advanced ccRCC between clusters. **(C)** Differential analysis of immune cell infiltration between two clusters using ssGSEA method. **(D)** Relationship between differences in PD-1 and CTLA-4 responsiveness between the two groups, based on the TCIA database. GP style: *: p< 0.05; **: p < 0.01; ***:p<0.001; **** p<0.0001. n.s. = no significance.

To explore potential biological functions and features between two clusters, we identified DECRGs between clusters by *limma* R package and performed functional enrichment using ClueGO plugin. We found that patients in Cluster 1 was significantly associated with Platelet Activation, Blood Coagulation, and Smooth muscle cell migration, while patients in Cluster 2 were significantly correlated with Complement and coagulation cascade and Negative regulation of low-density lipoprotein (**Figure 4A**). Subsequently, we explored the sensitivity of two clusters to drugs commonly used in clinically advanced ccRCC by pRRophetic. Compared to Cluster 2, patients in Cluster 1 were more sensitive to axitinib, pazopanib, Voninostat and sorafenib, which may be closely related to their enriched pathways (**Figure 4B**). Since ccRCC is a strongly immunogenic tumor, we then compared the immune cell infiltration of the two types of Clusters. Eosinophil and plasma cell infiltration was more pronounced in Cluster 1 patients, whereas T-cell infiltration was more pronounced in Cluster 2 patients, suggesting that there may be different sensitivities to immunotherapy between the two Clusters (**Figure 4C**). Also, we explored the responsiveness to immunotherapy in the TCIA database. Cluster 2 patients were more sensitive to CTLA4 or PD-1 and the combination of both (**Figure 4D**).

### Construction and validation of the diagnostic signature based on the DECRGs

**Figure 5:**
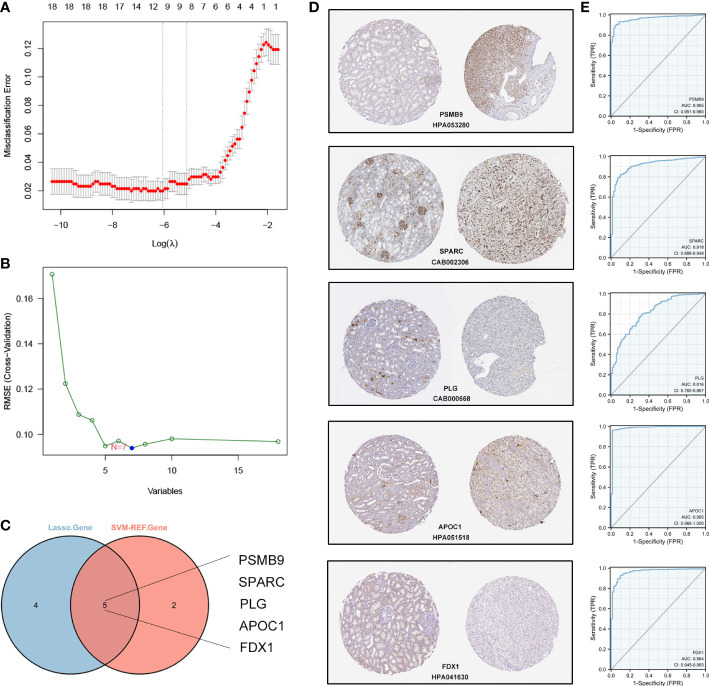
Establishment and validation of the diagnostic signature based on CRGs in TCGA cohort. **(A)** LASSO regression to identify signature genes in ccRCC and normal samples. **(B)** SVM-REF regression to identify significant CRGs in ccRCC and normal samples. **(C)** Venn diagram based on the intersection of the two algorithms with 5 genes. **(D)** The IHC-P images of 5 CRGs in HPA database. **(E)** ROC curves of 5 signature genes for predicting diagnostic value in TCGA cohort.

Based on the 16 hub DECRGs, we wanted to know if these genes could be used as markers for the diagnosis of ccRCC. We screened 9 and 7 CRGs using LASSO and SVM-REF regression analyses, respectively (**Figure 5A, B**). We then selected the intersecting genes of the two methods as diagnostic signature, including PSMB9, SPARC, PLG, APOC1, and FDX1 (**Figure 5C**). In the HPA database, we observed the protein expression of these five genes by immunohistochemical data. Compared to normal kidney tissues, PSMB9, SPARC and APOC1 expression levels were upregulated in ccRCC, while FDX1 and PLG expression levels were downregulated in ccRCC (**Figure 5D**). Afterwards, we evaluated the predictive diagnostic value of the diagnostic signature in the TCGA cohort, and the results showed a good predictive value in ccRCC (**Figure 5E**).

### Construction and validation of the prognostic signature based on the prognostic CRGs

**Figure 6:**
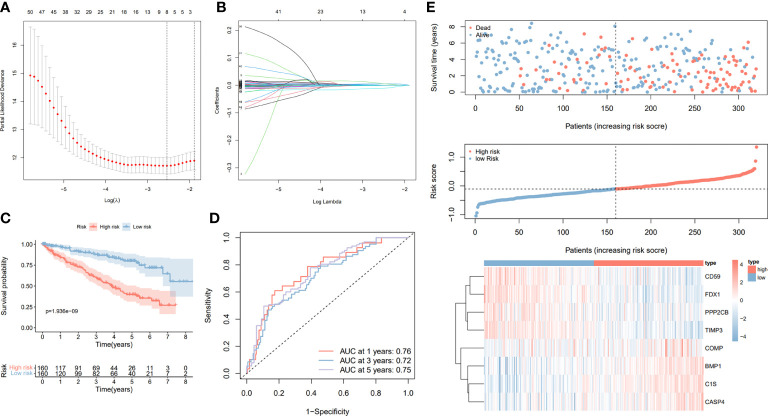
Construction and validation of the prognostic signature based on the CRGs. **(A)** Cross-validation of the parameter selection in the LASSO regression. **(B)** LASSO regression of the 8 CRGs related to the OS. **(C)** Kaplan–Meier survival curves between high- and low-risk groups. **(D)** The AUC value of ROC curves of prognostic signature for predicting 1-year, 3-year, and 5-year OS in the TCGA cohort. **(E)** Signature gene expression patterns and the distribution of survival status and risk score in the TCGA training cohort.

To further investigate the prognostic value of CRGs, we conducted a univariate Cox regression of 279 CRGs in relation to OS. A total of 99 prognostic CRGs with P < 0.01 were identified. We then build the prognostic signature by LASSO regression (**Figure 6A, B**). We then calculated the riskScore by using the formula in the material part of the method. Based on the calculated median riskScore cut-off, patients in training and validation cohort were divided into the high- and low-risk groups. Furthermore, the Kaplan–Meier log-rank test and the time-dependent ROC curve were used to evaluate the predictive ability and accuracy of the prognostic signature. The outcome of the Kaplan–Meier log-rank test showed that the high-risk group had a significantly worse OS compared with the low-risk group in the TCGA training set (**Figure 6C**), TCGA validation set (**Figure S2A**), and E-MTAB validation set (**Figure S2C**). Meanwhile, the time-dependent ROC curve proved the 1-year, 3-year, and 5-year predictive accuracy of the signature for OS (**Figures 6D**, **S2B, S2D**). In addition, the risk score distribution, survival status, and expression of CRGs from the signatures are shown in the TCGA training cohort, TCGA validation cohort, and E-MTAB validation cohort (**Figures 6E**, **S2E, S2F**). Also, as shown in **Figure S3**, we compared our signature with previously published signatures in the TCGA dataset, and the results showed that our signature had better predictive performance, especially for 5-year survival (23, 24).

### Establishment and evaluation of the nomogram

**Figure 7:**
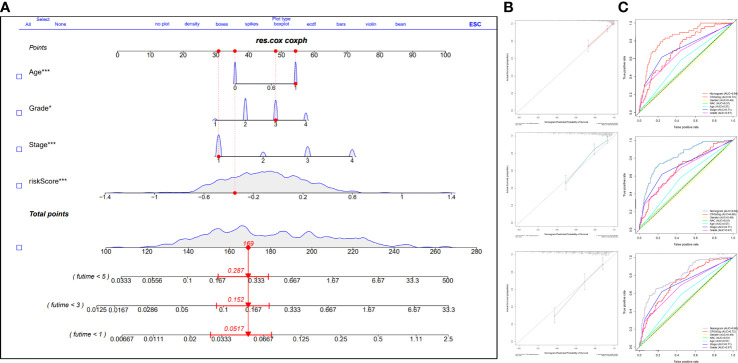
Construction and validation of the prognostic nomogram integrating prognostic signature and clinical parameters. **(A)** The nomogram based on the significant clinical parameters and risk signature. **(B)** Calibration curves of the nomogram for 1-, 3-, and 5-year survival prediction. **(C)** The AUC value of ROC cruves of the nomogram, risk signature, and clinical parameters. GP style: *: p< 0.05; ***:p<0.001.

To predict the prognosis of ccRCC patients more accurately, we identified independent risk factors affecting OS by univariate and multivariate regression analysis (**Table 1**). The outcomes showed that the Stage, Grade, Age, and Riskscore could be the independent factors for OS of ccRCC patients (**Table 1**). Combining the calculated riskScore and independent clinical parameters, we established the nomogram with a C-index 0.773 (**Figure 7A**). Then, we performed the calibration curves to verify the predictive efficacy of Nomogram for 1-year, 3-year and 5-year OS (**Figure 7B**). We then confirmed the prognostic value of this Nomogram over the TNM Staging system or the Grade system for ccRCC patients by using multiple ROC curves (**Figure 7C**).

**Table 1:** Univariate and multivariate Cox analyses of clinical parameters and risk signature.

	Univariate analysis		Multivariate analysis	
Parameters	HR (95%CI)	ρ value	HR (95%CI)	ρ value
Gender	0.930(0.672, 1.287)	0.663	0.865(0.624, 1.200)	0.387
Stage	1.899(1.656, 2.178)	<0.001	1.566(1.333, 1.838)	<0.001
Grade	2.434(1.959, 3.024)	<0.001	1.354(1.052, 1.742)	0.019
Age	1.774(1.285, 2.448)	<0.001	1.813(1.303, 2.520)	<0.001
CRGSig	6.800(4.322, 10.698)	<0.001	3.534(2.043, 6.111)	<0.001
NAC	1.868(0.952, 3.665)	0.069	0.993(0.623, 1.201)	0.985

### Further exploration of the significant CRGs FDX1

**Figure 8:**
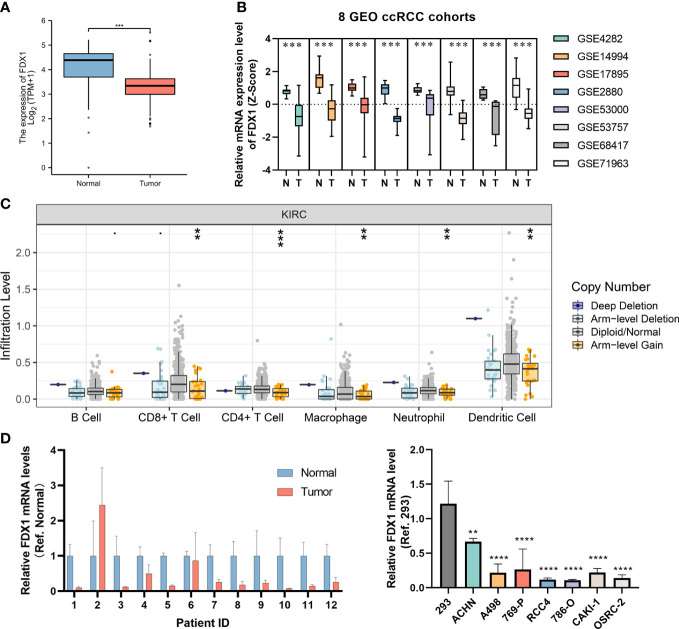
Further exploration of the FDX1. **(A)** The bar plot of the FDX1 mRNA expression between ccRCC and normal tissues in TCGA and GTEx database. **(B)** Analysis of 8 GEO datasets regarding FDX1 mRNA expression in ccRCC and normal samples. **(C)** The association of FDX1 CNV status with immune infiltration abundance in ccRCC was evaluated using TIMER database. **(D)** The validation of FDX1 mRNA expression level in our clinical specimens and cell lines. GP style: **: p < 0.01; ***:p<0.001; **** p<0.0001.

Since the FDX1 was shown to be important in both diagnostic and prognostic signature, we further explored the potential value of FDX1 in ccRCC plus GTEx normal kidney mRNA expression data (**Figure 8A**). We then compared the expression levels of FDX1 in ccRCC and normal kdiney tissues in 8 GEO cohorts and found that the FDX1 were significantly upregulated in normal tissues (**Figure 8B**). Subsequently, based on the Timer database, we found that FDX1 copy number alterations significantly affected the level of infiltration of several major immune cells (**Figure 8C**). We verified FDX1 expression levels in our own clinical samples and cell lines, and consistently with the results in the online database, FDX1 was significantly downregulated in ccRCC (**Figure 8D**).

## Discussion

Malignant tumors affect the hemostatic system, while abnormal coagulation states have been observed frequently in patients with malignant tumors (25). For ccRCC patients, venous tumor thrombosis often implies poor prognosis (26). Currently, ccRCC patients with venous system involvement have a high risk of tumor recurrence even after the tumor thrombus has been successfully eliminated (27). This suggests that coagulation abnormalities might serve as a critical factor for the prognosis of patients with ccRCC. Indeed, we found a more extensive hypercoagulable state in advanced ccRCC patients in our clinical data. In addition to that, former studies have shown that coagulation status significantly affects the immune function (28, 29). This suggested that coagulation was likely to be associated with immunotherapy effects. As a strong immunogenic tumor, immunotherapy for ccRCC patients holds great promise. The function of coagulation in predicting the prognosis of ccRCC as well as the effect of immunotherapy remains to be explored.

First, we found in our clinical data that patients with high stage and grade were more likely to occur coagulation system dysfunction. The phenomenon is widespread in a variety of advanced tumors (30, 31). In ccRCC, presence of inferior vena cava tumor thrombosis often indicates poor prognosis. Furthermore, previous studies reported that extensive infiltration of exhausted CD8 T cells were gathered in the tumor thrombosis, which might influence the effect of ICIs (32). Meanwhile, drugs commonly used in advanced kidney cancer, such as sunitinib, can affect the coagulation system and platelet formation (33). These findings suggested that exploring the CRGs could benefit the diagnostic detection, prognostic evaluation, and personalized treatment of ccRCC patients. Based on these conjectures, we first performed molecular subtyping of ccRCC patients based on the CRGs. Indeed, Patients from different clusters showed markedly diverse therapeutic effects to CTLA4, sunitinib, and so on. This suggests that molecular subtyping of ccRCC using CRGs could contribute to more precise personalized treatment.

Next, we constructed the diagnostic and prognostic signature using the SVM-REF and LASSO methods. Diagnosis-related signature genes included APOC1, PSMB9, SPARC, PLG and FDX1. CD59, FDX1, PPP2CB, TIMP3, COMP, BMP1, C1S, and CASP4 were identified as prognostic signatures. Some genes in signature have been extensively reported in previous studies. APOC1 was mainly expressed in macrophages and closely associated with immune cell infiltration in RCC. Macrophages with high APOC1 expression promote RCC metastasis by secreting CCL5 (34). Recent study suggested that APOC1 was correlated with ferroptosis, which may be influenced by lipid metabolism through its apolipoprotein function. Expression levels of PSMB9 were significantly up regulated in patients who continued to benefit from ICIs, suggesting that it could be a target for the assessment of therapeutic efficacy of ICIs, in agreement with our analysis (35). The expression of SPARC was increased in all subtypes of RCC and positively correlated with RCC staging and grading (36). Knockdown SPARC significantly inhibit RCC cell invasion and metastasis *in vitro* and *in vivo*. In addition, the expression of SPARC was negatively correlated with the overall survival and disease-free survival of RCC patients, indicating that SPARC is a valid prognostic marker for the survival of RCC patients (37). Plasminogen (PLG) encode the plasminogen, which circulates in blood plasma as an inactive zymogen and is converted to the active protease, plasmin by several plasminogen activators (38). PLG was over-expressed in HBV positive hepatocellular carcinoma tissues and cells. PLG silencing promoted HBV-HCC cell apoptosis *in vitro* and suppressed the growth of HBV-induced HCC xenografts *in vivo* both through inhibiting HBV replication (39). PLG, as a prognosis-related gene, has been applied to construct prognosis-related signature in a variety of tumors (40–42). PPP2CB is the catalytic subunit β isoform of phosphatase 2A (PP2A). PP2A regulates T cell activation, which plays an important role in immune homeostasis (43). CD59 has been identified as a glycosylphosphatidylinositol-anchored membrane protein that acts as an inhibitor of the formation of the membrane attack complex to regulate complement activation (44). Recent studies have shown that CD59 is highly expressed in several cancer cell lines and tumor tissues. CD59 also regulates the function, infiltration and phenotypes of a variety of immune cells in the tumor microenvironment (45). CD59 is up-regulated on activated CD4(+) T cells and serves to down-modulate their activity in response to polyclonal and Ag-specific stimulation (46). CD59 is expressed in renal tumor cells and proximal tubular epithelial cells, which plays a role in preventing complement-mediated lysis of these cells (47). TIMP3 is considered to be an anti-angiogenic factor. In ccRCC, the expression of TIMP3 is associated with the patient’s prognosis. Furthermore, in high-grade renal cell carcinoma tumors, TIMP3 mRNA levels were significantly lower (48). COMP has a protective effect on cyclosporine-induced kidney injury (49) and can improve renal fibrosis (50). The role of COMP in ccRCC needs further investigation. Bone morphogenetic proteins (BMP) family is a group of proteins found in recent years that are related to the pathogenesis of a variety of cancers (51). The high expression of BMP1 is a poor prognostic factor in patients with renal clear cell carcinoma, and knocking down BMP1 inhibits the proliferation and invasion of renal clear cell carcinoma *in vitro* and *in vivo* (52). C1S has a dual role in promoting ccRCC, and renal tumors expressing high levels of C1S show high infiltration of macrophages and T cells (53). Studies have shown that abnormal activation of C1S contributes to the development of autoimmune and infectious diseases. In addition, the overexpression of C1S may be a new escape mechanism to promote tumor progress (54). CASP4, as a gene related to cell apoptosis, is differentially expressed in a variety of tumors, and can be used to predict the prognosis of tumor patients (55, 56). CASP4 is highly expressed in ccRCC, which is correlated with high pathological scores, poor prognosis and expression level of infiltrating immune cells (57). FDX1, which is more prominent in both diagnostic and prognostic signatures, has not been previously reported, but there has been a significant increase in studies about FDX1 this year, and the mechanism remains to be explored (58, 59).

The present study has some shortcomings. Firstly, our study is based on various published databases, and it was difficult to completely batch effect and remove the background differences between databases and sequencing platforms. Second, the small sample size and lack of multi-omics data limited the accuracy of molecular subtyping of ccRCC patients, which also inevitably led to the accuracy of diagnostic and prognostic signature. Finally, an important gene FDX1 identified in this study, was only validated for its expression in clinical specimens and cell lines, subsequent validation of its function *in vitro* and *in vivo* assays was needed.

## Conclusion

In conclusion, using single cell and RNAseq data, we preliminarily demonstrated the prognosis and individualized treatment value of CRGs in ccRCC. Different immune states and drug responses were revealed by typing TCGA patients with CRGs, which is very important for individualized treatment of ccRCC patients. On this basis, we build the diagnostic signature, prognostic signature and nomogram based on CRGs, which can accurately screen patients with ccRCC and predict the prognosis of patients with ccRCC. Finally, we verified the vital FDX1 in our clinical samples and cell lines, and further experiments need to be carried out in the future.

## Data availability statement

The datasets presented in this study can be found in online repositories. The names of the repository/repositories and accession number(s) can be found in the article and **Supplementary Material**.

## Ethics statement

The studies involving human participants were reviewed and approved by Ethics committee of Yangzhou University. The patients/participants provided their written informed consent to participate in this study.

## Author contributions

YL, CZ, and ZZ conceptualized and designed the study. SZ, ZZ, GY, and TT wrote the article. GY, XJ, and TT collected and analyzed the data. GY, XJ, and TT carried out the experiments. GY, XJ, and TT contributed equally to this work. All authors contributed to the article and approved the submitted version.

## Supplementary material

Supplementary Figure 1 Differences in fibrinogen, PTR and PTA among patients with ccRCC of different stages and grades.

Supplementary Figure 2 (**A**, **B**) K-M plot and ROC curve for TCGA internal validation cohort. **(C)** The distribution, survival status, and gene heatmap of the prognostic signature for TCGA internal validation cohort. (**D**, **E**) K-M plot and ROC curve for E-MTAB external validation cohort. **(F)** The distribution, survival status, and gene heatmap of the prognostic signature for E-MTAB external validation cohort.

Supplementary Figure 3 The AUC value of ROC curves of prognostic signature in this study and published articles for predicting 1-year, 3-year, and 5-year OS in the TCGA cohort.
