# Supplementary figures and images for: Integrated analysis to identify the prognostic and immunotherapeutic roles of coagulation-associated gene signature in clear cell renal cell carcinoma
Source: Front Immunol. 2023 Mar 17;14:1107419. doi: 10.3389/fimmu.2023.1107419 (PMC10063824; doi:10.3389/fimmu.2023.1107419)

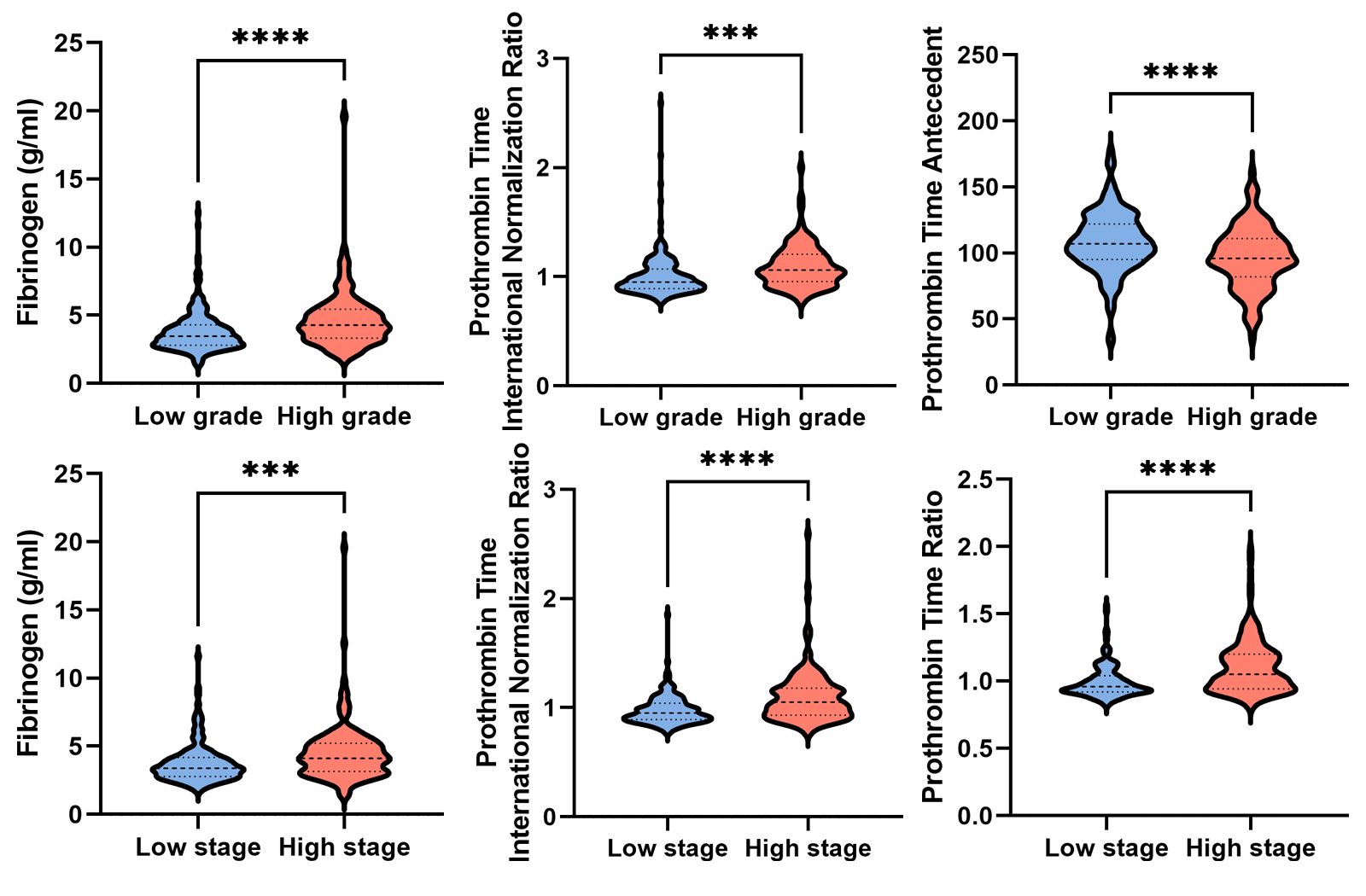

Supplement: Supplementary Figure 1 — Differences in fibrinogen, PTR and PTA among patients with ccRCC of different stages and grades. [file DataSheet_1.zip › Supplementary Figure S1.tif]

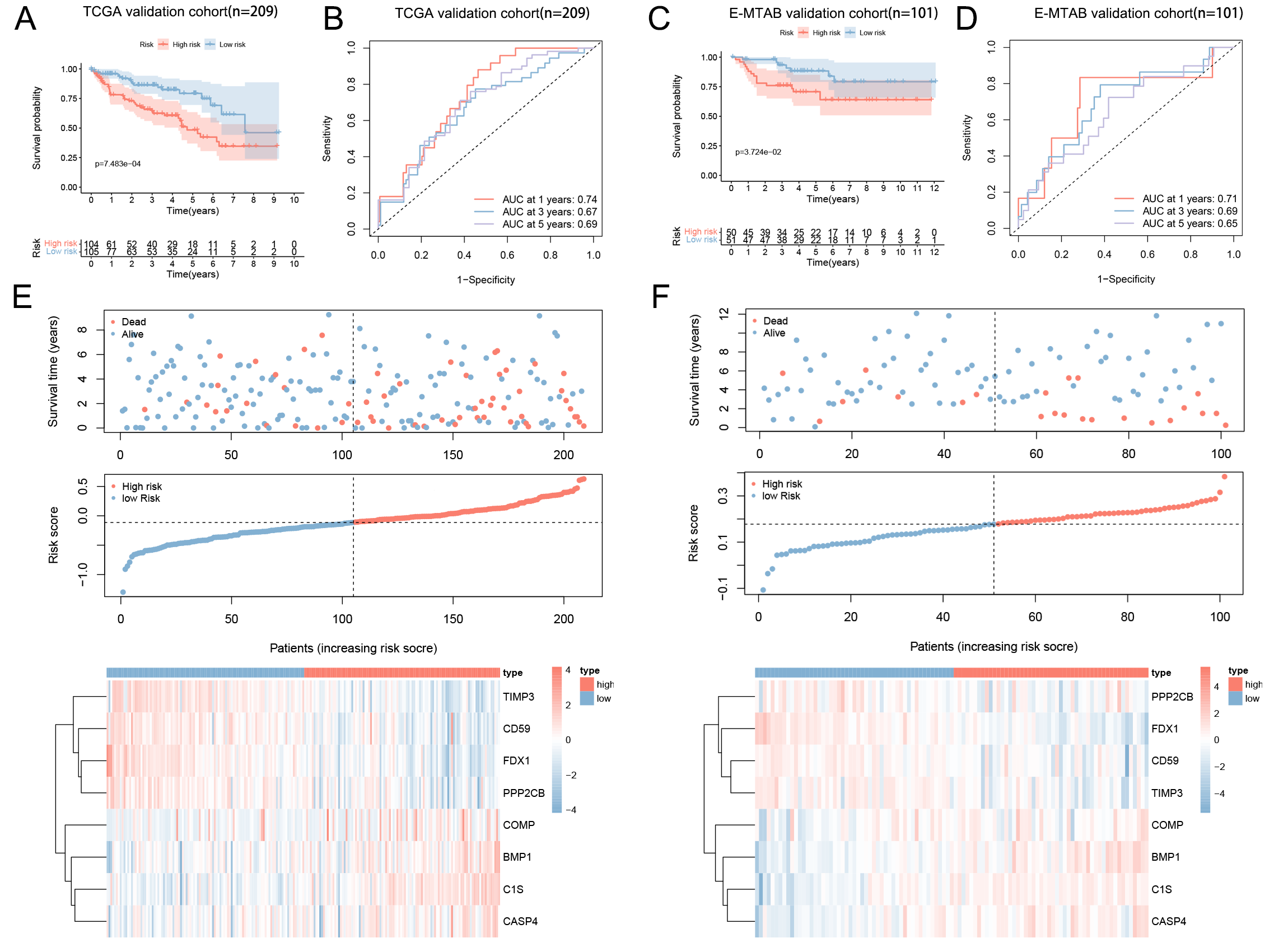

Supplement: Supplementary Figure 1 — Differences in fibrinogen, PTR and PTA among patients with ccRCC of different stages and grades. [file DataSheet_1.zip › Supplementary Figure S2.tif]

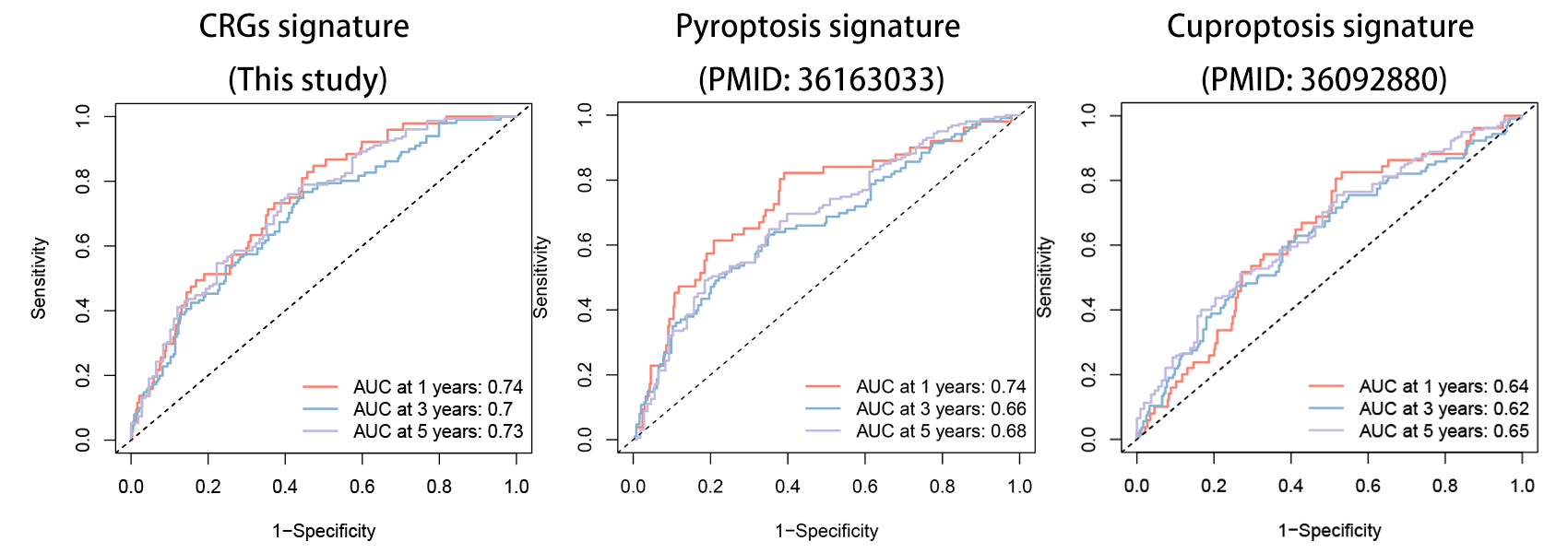

Supplement: Supplementary Figure 1 — Differences in fibrinogen, PTR and PTA among patients with ccRCC of different stages and grades. [file DataSheet_1.zip › Supplementary Figure S3.tif]
